# *NOD2* Polymorphisms and Their Association with Colorectal Cancer Risk: An Updated Systematic Review and Meta-Analysis

**DOI:** 10.3390/cancers17121999

**Published:** 2025-06-15

**Authors:** Mohamad Ayub Khan Sharzehan, Hilary Sito, Md Asiful Islam, Rahman Jamal, Shing Cheng Tan

**Affiliations:** 1 UKM Medical Molecular Biology Institute, Universiti Kebangsaan Malaysia, Cheras 56000, Kuala Lumpur, Malaysia; 2 Department of Biomedical Science and Physiology, School of Pharmacy and Life Sciences, Faculty of Science and Engineering, University of Wolverhampton, Wolverhampton WV1 1LY, UK

**Keywords:** NOD2, colorectal cancer, genetic polymorphism, risk, meta-analysis

## Abstract

**Background:** Nucleotide-binding oligomerization domain-containing protein 2, encoded by *NOD2*, can trigger chronic gut inflammation that leads to colorectal cancer (CRC). However, studies that have investigated the association of *NOD2* polymorphisms and CRC susceptibility have produced inconsistent findings. To clarify this relationship, a meta-analysis was conducted to integrate data from previous studies to achieve a more precise evaluation of the risk association. **Methods:** PubMed, Scopus, and Web of Science databases were systematically searched to identify relevant studies on the association of *NOD2* polymorphisms with CRC risk. Genetic risk association was quantitatively assessed under five genetic models: homozygous, heterozygous, dominant, recessive, and allele. Thirteen studies, comprising 5,013 cases and 4,463 controls, were included in this study. Four *NOD2* polymorphisms were investigated in these studies, namely rs2066842, rs2066844, rs2066845, and rs2066847. **Results:** Of these, only rs2066845 and rs2066847 were found to be significantly associated with increased CRC risk (rs2066845, heterozygous OR = 1.544, 95% CI = 1.014–2.349, P = 0.043; dominant OR = 1.561, 95% CI = 1.035–2.354, P = 0.034; allele OR = 1.572, 95% CI = 1.040–2.375, P = 0.032; rs2066847, heterozygous OR = 1.321, 95% CI = 1.060–1.647, P = 0.013; dominant OR = 1.402, 95% CI = 1.147–1.713, P = 0.001; allele OR = 1.345, 95% CI = 1.088–1.663, P = 0.006). **Conclusions:** In conclusion, the *NOD2* rs2066845 and rs2066847 polymorphisms are associated with an increased risk of CRC and may potentially serve as predisposition biomarkers for the cancer.

## Simple Summary

The NOD2 protein plays a crucial role in regulating intestinal inflammation. The dysregulation of NOD2, often due to genetic variations (polymorphisms), has been implicated in chronic gut inflammation and, consequently, increased colorectal cancer (CRC) risk. However, prior research on the association between *NOD2* polymorphisms and CRC susceptibility has yielded inconsistent results. This meta-analysis aimed to synthesize the existing evidence to provide a more robust assessment of this association. Our findings indicate that two specific *NOD2* polymorphisms, rs2066845 and rs2066847, are significantly associated with an elevated risk of CRC. These insights may contribute to the identification of individuals predisposed to CRC, thereby facilitating early detection and potentially guiding personalized preventive strategies in clinical practice.

## 1. Introduction

Colorectal cancer (CRC) is one of the most commonly diagnosed cancers and a leading cause of cancer-related deaths worldwide [1]. Although dietary and environmental factors have been well-established as major risk factors in the development of CRC, genetic factors, particularly genetic polymorphisms, have been shown to be an equally important element in determining individual susceptibility to the disease [2]. Therefore, identification of genetic polymorphisms associated with CRC may facilitate the early detection of at-risk individuals, which will allow preventive strategies to be taken before symptoms appear [3].

It has been known for some time that CRC may be preceded by inflammatory bowel disease (IBD), which includes Crohn’s disease (CD) and ulcerative colitis (UC) [4]. Therefore, genes associated with IBD pathogenesis are ideal candidates for biomarker-based studies on CRC. One such gene is *nucleotide-binding oligomerization domain 2* (*NOD2*) that is located on chromosome 16q12 [5]. NOD2 is one of the most important members of the caspase activation and recruitment domain subfamily capable of recognizing the conserved muramyl dipeptide (MDP) structure present in virtually all bacterial types [6,7]. The gene encodes an intracellular protein that belongs to the Nod-like receptors (NLRs), which contain a C-terminal sensor domain, a central nucleotide-binding oligomerization domain, and an N-terminal effector domain [8]. 

The NOD2 protein is directly involved in the regulation of the immune response through the activation of nuclear factor-kappa B (NF-κB) via the RIP2/IKK pathway [9]. In response to the presence of MDP, NOD2 interacts with RIP2 kinase to activate NF-κB and mitogen-activated protein kinase, leading to the transcription of proinflammatory mediators [10]. Mutations in *NOD2* result in increased NF-κB activity, a phenomenon observed in various human malignancies such as colorectal, thyroid, breast, and lung cancers [11,12]. *NOD2* also routinely stimulates host defense when it detects elevated levels of MDP following partial degradation of bacterial peptidoglycan [13]. In addition, the protein is actively involved in the recycling and degradation of the bacterial cell components through autophagy [2]. As the role of the gut microbiome in the development of CRC becomes increasingly clear, the ability of NOD2 to modulate bacterial growth suggests involvement of the protein in carcinogenesis.

Polymorphisms in *NOD2* may affect the functionality of its protein product and thus the risk for various diseases. There are four major *NOD2* single nucleotide polymorphisms that have been extensively studied, namely rs2066842 (conventionally known as Pro268Ser), rs2066844 (conventionally known as Arg702Trp), rs2066845 (conventionally known as Gly908Arg), and rs2066847 (conventionally known as 3020insC/Leu1007fsX1008). These polymorphisms are located in the coding region of *NOD2* and cause a change in the amino acid sequence of the protein product, which subsequently affects its expression and normal function [8]. For this reason, these polymorphisms have been found to be associated with a higher risk of CD [14,15,16]. In CRC, however, the association between *NOD2* polymorphisms and the risk of developing cancer remains controversial. For example, although [5,17,18,19] reported that there was no apparent association between the four polymorphisms and the risk of CRC, a few other studies demonstrated a significant association between the polymorphisms and CRC risk [11,20,21,22]. These discrepancies could be attributed to the different genetic and environmental backgrounds of the study subjects in different studies. To address these inconsistencies, a meta-analysis was conducted by [8] to combine the results of studies published before July 1, 2013. Nevertheless, numerous newer studies have been published more recently, and the inclusion of these more recent studies could potentially lead to a different conclusion. Thus, in this work, we performed an updated meta-analysis to investigate the association between *NOD2* polymorphisms and CRC risk.

## 2. Materials and Methods

### 2.1. Literature Search Strategy and Study Selection

This systematic review was conducted in accordance with the Preferred Reporting Items for Systematic Reviews and Meta-Analyses (PRISMA) 2020 guidelines. A thorough literature search was conducted on the Web of Science, PubMed, and Scopus databases on 28 May 2025. No language limitations were applied during the search. The following terms were used: “NOD2” AND “polymorphism” AND “colorectal cancer”. Two investigators independently screened the studies for inclusion in the meta-analysis, based on the following eligibility criteria: (1) assessing the associations between *NOD2* rs2066842, rs2066844, rs2066845, and rs2066847 polymorphisms and CRC risk; (2) case–control study; and (3) providing sufficient data for the odds ratio (OR) and 95% confidence interval (CI) calculation. Non-original studies (including review, editorials, and letters to the editor) were excluded. Furthermore, the reference lists of the included studies were also screened for additional potentially relevant articles. If two or more publications were available for the same population, the one containing either the highest number of samples or the latest dataset was selected. No prospective registration was carried out for this review.

### 2.2. Data Extraction and Quality Assessment

For each eligible study, the following information was extracted independently by the two investigators in an Excel sheet: name of the first author, publication year, country, ethnicity, the genotype, and allele frequencies of the four *NOD2* polymorphisms, and deviation from the Hardy–Weinberg equilibrium (HWE). The ethnicity of the study populations was categorized as Caucasian, African, Asian, and others. The quality of the included studies was evaluated based on the Modified Newcastle-Ottawa Scale for Case-Control Studies of Genetic Association [23] independently by the two investigators. Studies with a rating of 5 stars or above were considered as having a high methodological quality. 

### 2.3. Statistical Analysis

The pooled OR and the 95% CI were calculated to evaluate the associations of *NOD2* rs2066842, rs2066844, rs2066845, and rs2066847 polymorphisms with CRC risk. Meta-analysis was performed only when data from at least three studies were available. For all calculations, wild-type genotype and/or allele were used as the reference group. The significance of the overall OR was determined using the Z-test. Cochran’s Chi-squared-based Q test and I2 test were used to assess the presence of statistical heterogeneity among the studies. If significant heterogeneity was present (as indicated by an I2 value ≥ 50% and P < 0.10, the random-effects model (the DerSimonian–Laird method) was used to calculate the pooled OR. Meanwhile, a fixed-effects model (the Mantel–Haenszel method) was used to calculate the pooled OR when there was no apparent between-study heterogeneity. The stability of the test results was determined using sensitivity analysis by excluding one study at a time. Subgroup analysis by ethnicity was also performed. In addition, funnel plots, Egger’s test, and Begg’s test were applied to examine the presence of publication bias. All statistical analyses were performed using Stata version 18.0 software (StataCorp, College Station, TX, USA), by assuming P < 0.05 as statistically significant, unless otherwise stated.

## 3. Results

### 3.1. Characteristics of Studies

**Figure 1:**
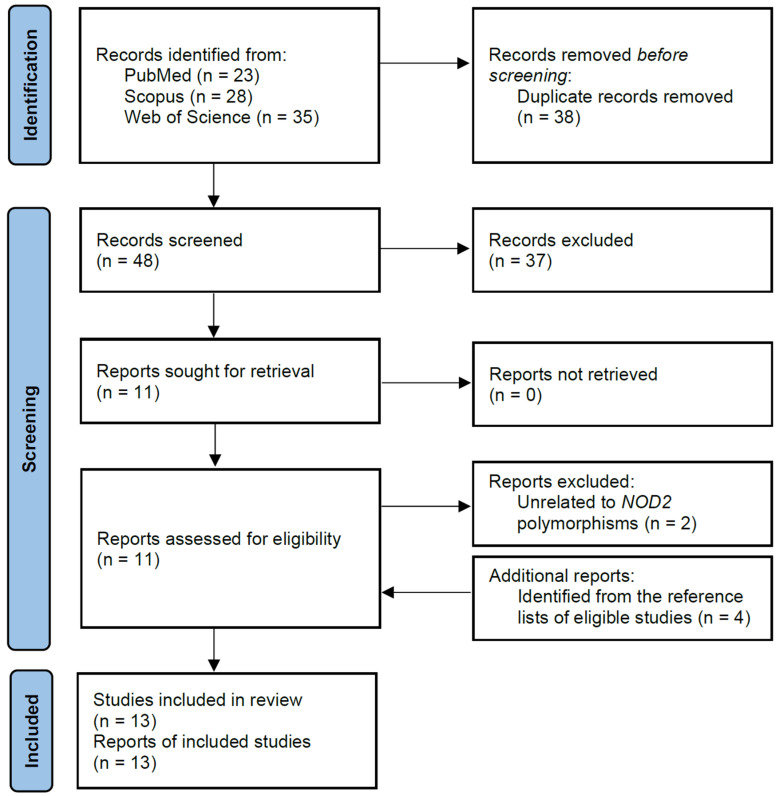
Flow chart of selection of eligible studies.

Using the search strategy, a total of 86 records were found in the PubMed, Scopus, and Web of Science databases. Of these, 38 duplicated records were removed, leaving 48 articles that were screened based on titles and abstracts. After reviewing the titles and abstracts, 11 articles were determined as being potentially relevant and were further assessed for eligibility. Based on the eligibility criteria, two articles were subsequently removed as they did not contain information on the polymorphisms of interest. After the screening process, four additional articles were identified from the reference lists of eligible studies and were included in this meta-analysis, resulting in a total of 13 studies comprising 9476 subjects (5013 cases and 4463 controls). The flow chart of the study selection process is summarized in Figure 1, and the characteristics of the included articles are shown in Table 1.

For rs2066842, there were three studies comprising a total of 314 cases and 513 controls that were eligible for meta-analysis. In addition, eight studies with 2718 cases and 2310 controls were included for investigating the association between the rs2066844 polymorphism and CRC risk. On the other hand, 7 studies with 2616 cases and 2045 controls were included for rs2066845, and 11 studies involving 3945 cases and 3690 controls were included for the rs2066847 polymorphism. These studies were conducted in Denmark, Northern Germany, Hungary, Poland, Romania, Malaysia, Tunisia, Finland, Greece, and New Zealand. The genotype distributions in the controls of all studies were consistent with the HWE, with the exception of Szeliga et al. [22] (for all the four polymorphisms studied), Mockelmann et al. [20] (for rs2066847), and Lau et al. [12] (for rs2066844, rs2066845, and rs2066847). All studies had high methodological quality as assessed using the Newcastle-Ottawa Scale (Table 2).

### 3.2. Quantitative Data Synthesis

The pooled association of the *NOD2* rs2066844, rs2066845, and rs2066847 polymorphisms with the risk of CRC is shown in Table 3, Table 4 and Table 5. Meta-analysis was not performed for rs2066842, as well as the homozygous and recessive models of the three aforementioned polymorphisms, because the number of included studies was too small to be analyzed after excluding studies with zero-count cells, i.e., studies where no events occurred in either the case or control group. Subgroup analysis by study quality was also not performed as all studies had high methodological quality. In addition, subgroup analysis by ethnicity was performed only for the Caucasian and “other ethnicity” subgroups, but not for the Asian subgroup, as there was only one study involving Asians.

**Figure 2:**
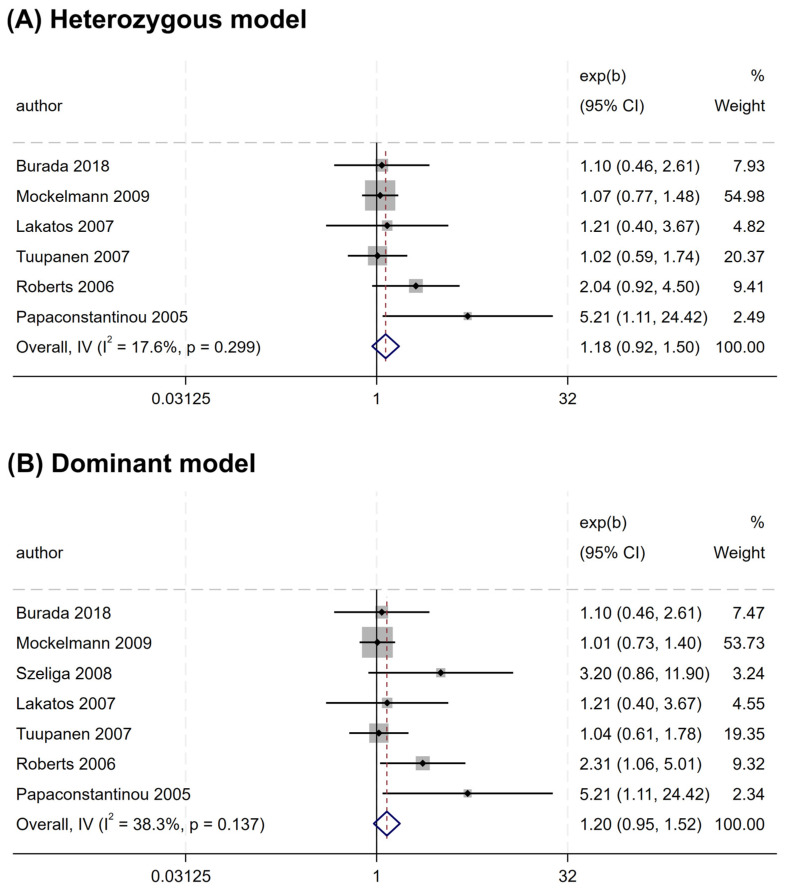

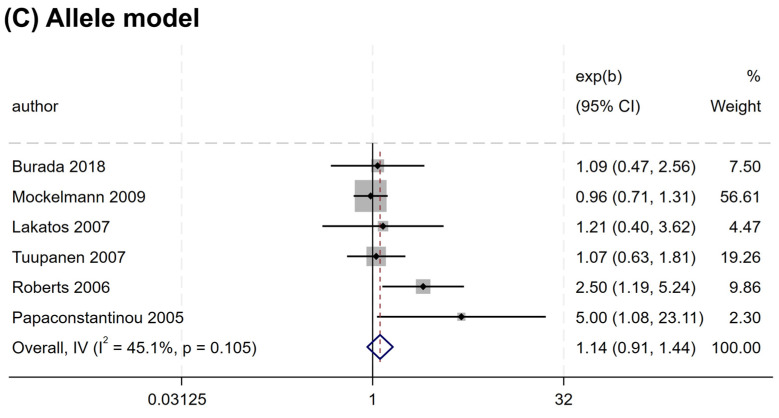
Forest plots of association between *NOD2* rs2066844 polymorphism and colorectal cancer risk. (**A**) Heterozygous model; (**B**) Dominant model; (**C**) Allele model.

Overall, no significant association was found between the rs2066844 polymorphism and the risk of CRC under all genetic models analyzed (heterozygous model, OR = 1.176, 95% CI = 0.922–1.501, P = 0.191; dominant model, OR = 1.253, 95% CI = 0.989–1.589, P = 0.062; allele model, OR = 1.243, 95% CI = 0.983–1.571, P = 0.069) (Table 3 and Figure 2). In the subgroup analysis based on ethnicity, rs2066844 was also not significantly associated with the risk of CRC in either the Caucasian or “other ethnicity” subgroups (P > 0.05).

**Figure 3:**
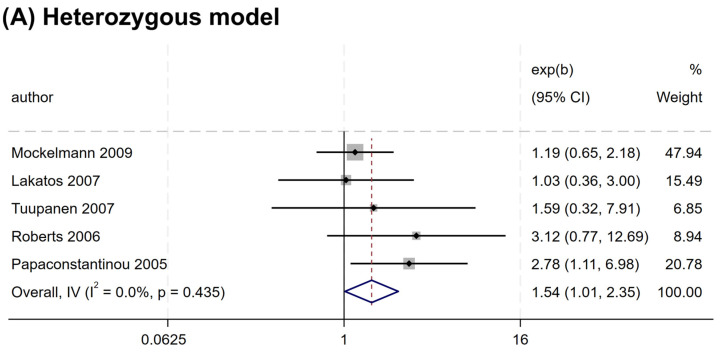

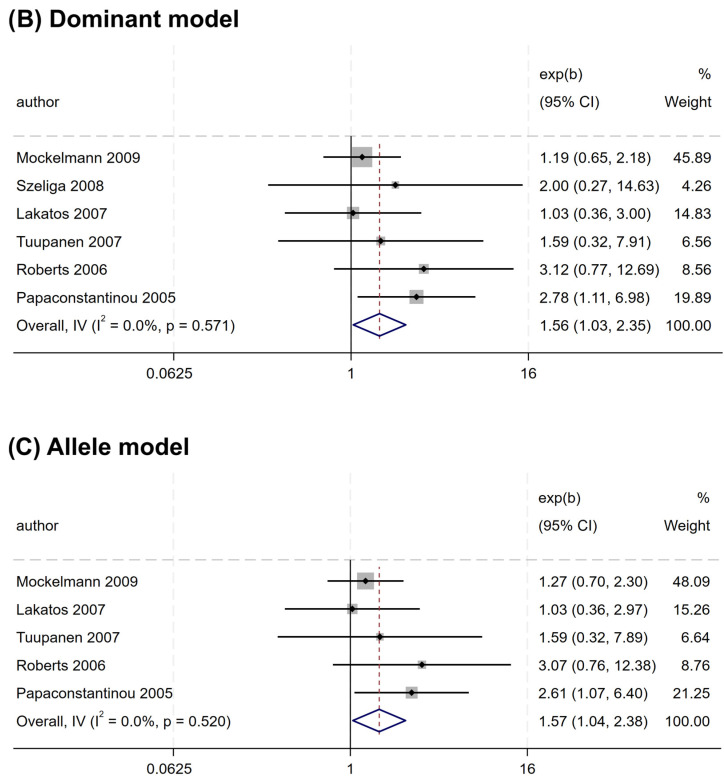
Forest plots of association between *NOD2* rs2066845 polymorphism and colorectal cancer risk. (**A**) Heterozygous model; (**B**) Dominant model; (**C**) Allele model.

Nevertheless, a significant association was observed for rs2066845 and rs2066847. An increased risk association was noted for rs2066845 (heterozygous model, OR = 1.544, 95% CI = 1.014–2.349, P = 0.043; dominant model, OR = 1.561, 95% CI = 1.035–2.354, P = 0.034; allele model, OR = 1.572, 95% CI = 1.040–2.375, P = 0.032) (Table 4 and Figure 3). Despite this, none of the subgroups showed a significant association (P > 0.05).

**Figure 4:**
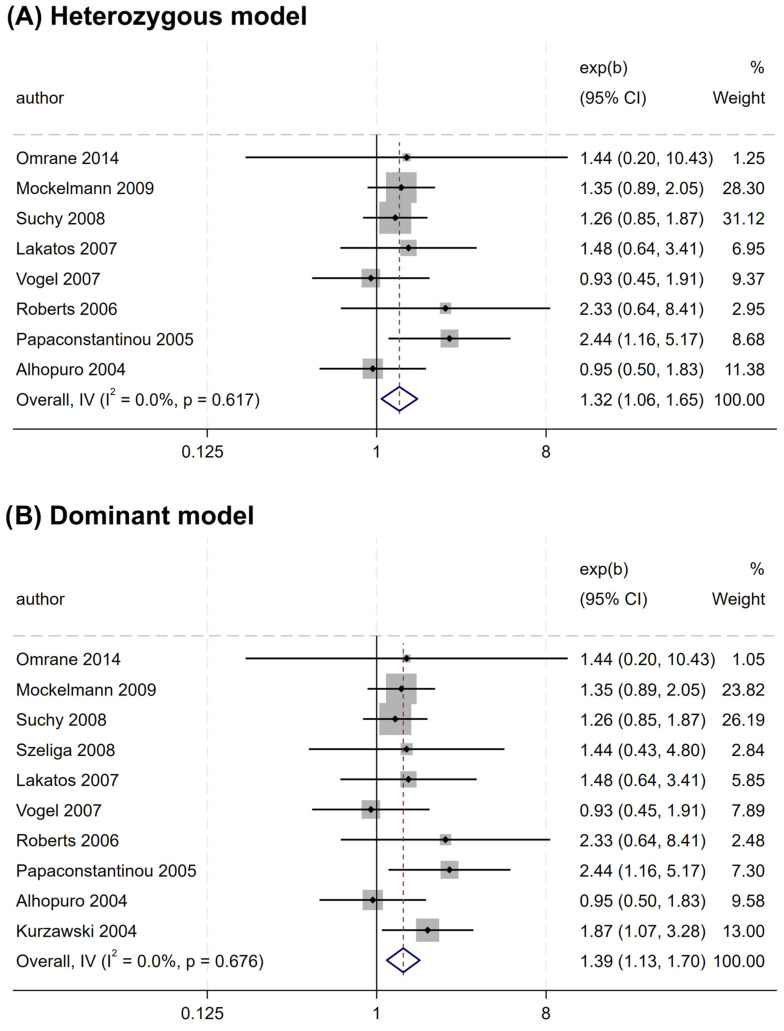

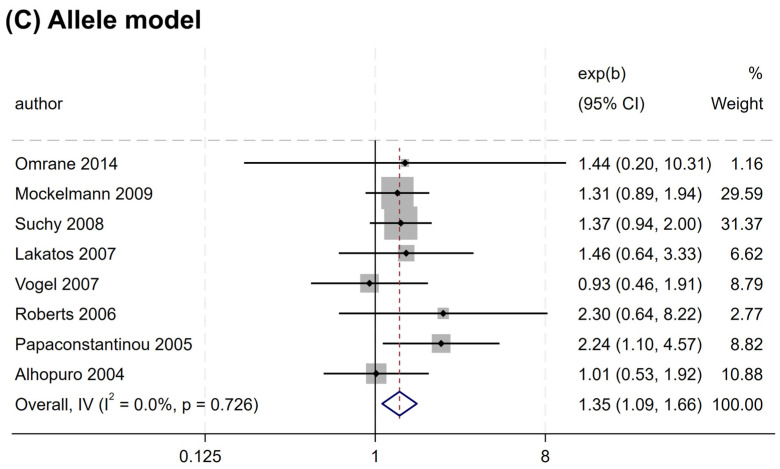
Forest plots illustrating the relationship between the *NOD2* rs2066847 variant and the risk of CRC. (**A**) Heterozygous model; (**B**) Dominant model; (**C**) Allele model.

Similarly, rs2066847 was also associated with an increased CRC risk (heterozygous model, OR = 1.321, 95% CI = 1.060–1.647, P = 0.013; dominant model, OR = 1.402, 95% CI = 1.147–1.713, P = 0.001; allele model, OR = 1.345, 95% CI = 1.088–1.663, P = 0.006) (Table 5 and Figure 4), and subgroup analysis revealed that the association was present only in subjects of “other ethnicity” (heterozygous model, OR = 1.343, 95% CI = 1.047–1.722, P = 0.020; dominant model, OR = 1.434, 95% CI = 1.149–1.788, P = 0.001; allele model, OR = 1.364, 95% CI = 1.076–1.729, P = 0.010), whereas no significant association was observed in the Caucasians (P > 0.05).

### 3.3. Sensitivity Analysis

Sensitivity analysis revealed that the omission of a few studies changed the association of rs2066844 with CRC from non-significant to significant under the dominant and allele models (Supplementary Information online). Likewise, but in the opposite direction, the omission of a few studies removed the significance of the CRC risk association conferred by rs2066845 (under all genetic models) and rs2066847 (under the heterozygous model). Nevertheless, this change was not surprising, given that the lower bound of the 95% CI was very near to the cutoff of 1.000.

### 3.4. Publication Bias

**Figure 5:**
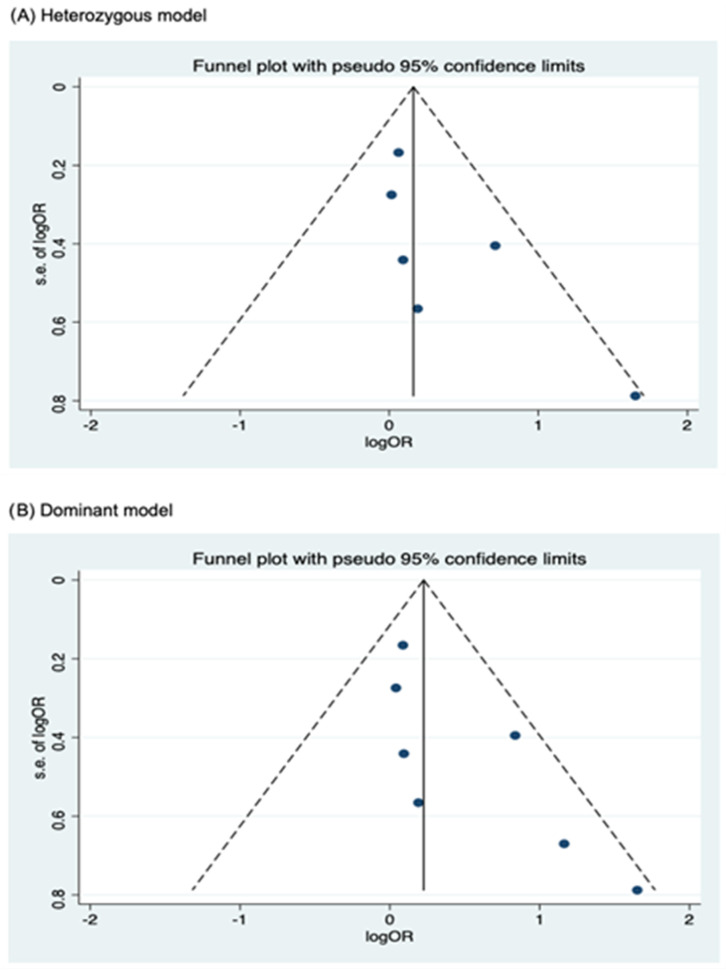

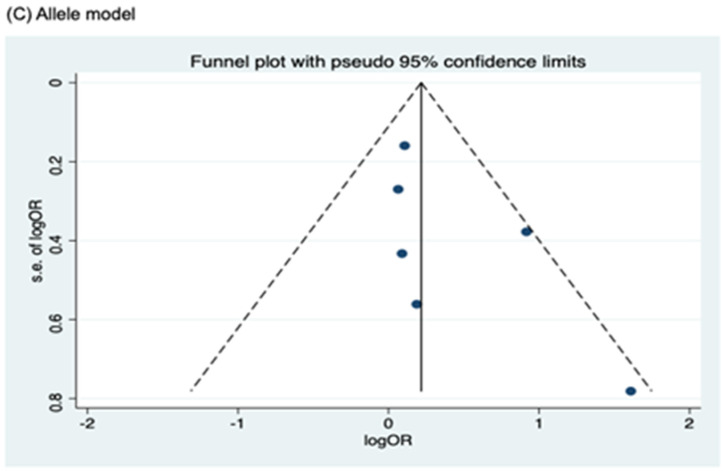
Funnel plot analysis to detect publication bias for *NOD2* rs2066844 polymorphism and colorectal cancer risk. (**A**) Heterozygous model; (**B**) Dominant model; (**C**) Allele model.

**Figure 6:**
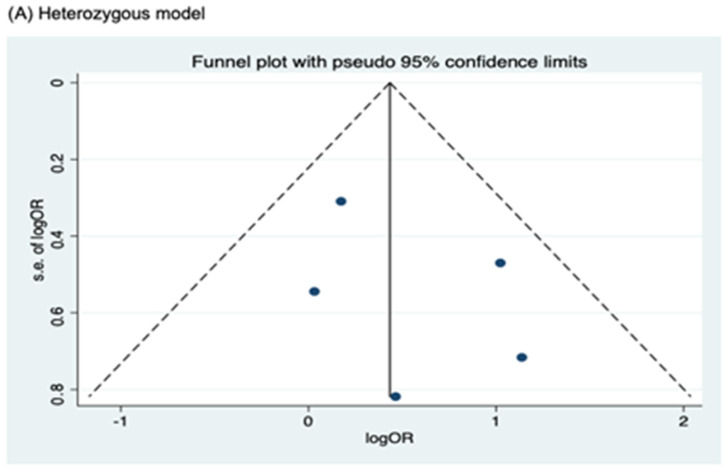

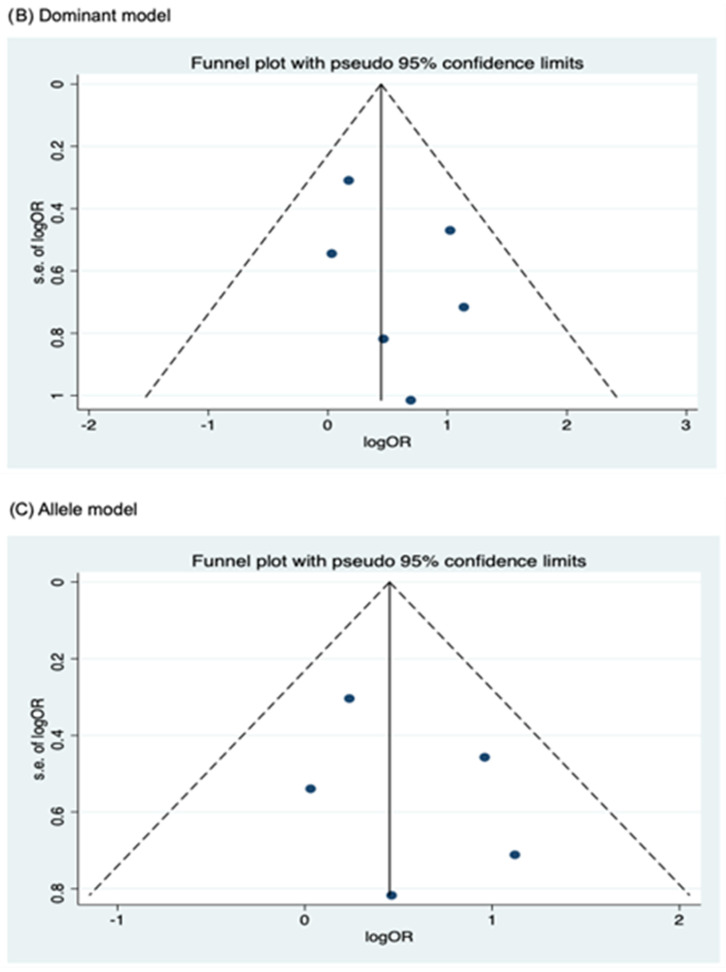
Funnel plot analysis to detect publication bias for *NOD2* rs2066845 polymorphism and colorectal cancer risk. (**A**) Heterozygous model; (**B**) Dominant model; (**C**) Allele model.

**Figure 7:**
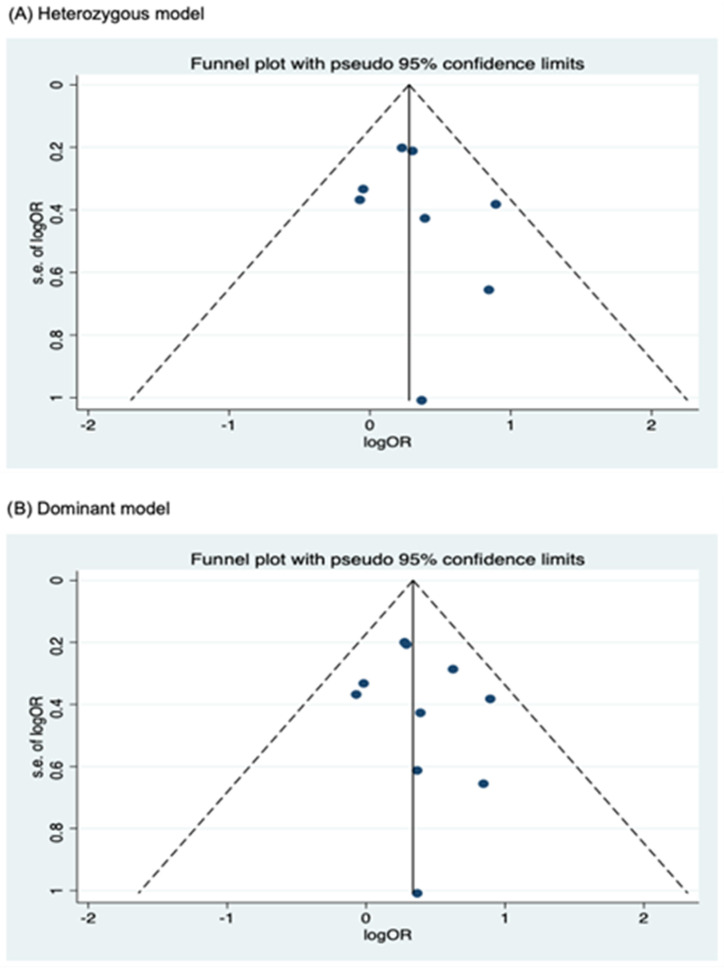

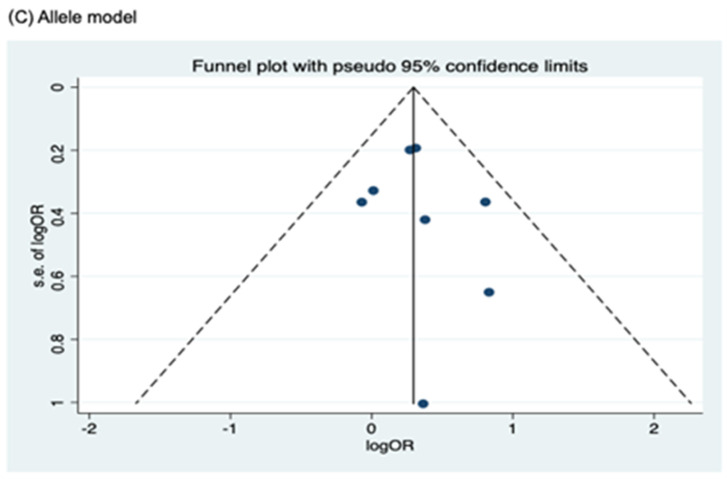
Funnel plot analysis to detect publication bias for *NOD2* rs2066847. (**A**) Heterozygous model; (**B**) Dominant model; (**C**) Allele model.

Funnel plots for assessing publication bias for rs2066844, rs2066845, and rs2066847 are shown in Figure 5, Figure 6, and Figure 7, respectively. Formal assessments with the Begg’s test revealed significant publication bias in the heterozygous and dominant models for rs2066844 (heterozygous model, P = 0.039; dominant model, P = 0.024), although no significant bias was detected with the Egger’s test (heterozygous model, P = 0.118; dominant model, P = 0.057). The discrepancy between Begg’s and Egger’s tests was not unexpected, as both tests have different sensitivity and statistical power, especially when the number of included studies is small. Performing both tests, especially when combined with visual inspection of the funnel plot, provides a more robust assessment, as each captures different aspects of potential publication bias and helps to validate findings through complementary statistical approaches. Nevertheless, a “trim and fill” analysis was performed for the two genetic models. In each model, one potentially missing study was identified and correction for the missing study did not significantly alter the results (heterozygous model, P = 0.310; dominant model, P = 0.110).

Meanwhile, no publication bias was observed for the allele model of rs2066844 (Begg’s test, P = 0.091; Egger’s test, P = 0.179), and for all genetic models of rs2066845 (heterozygous model, Begg’s test, P = 0.624, Egger’s test, P = 0.430; dominant model, Begg’s test, P = 0.573, Egger’s test, P = 0.371; allele model, Begg’s test, P = 0.624, Egger’s test, P = 0.484), as well as rs2066847 (heterozygous model, Begg’s test, P = 0.322, Egger’s test, P = 0.503; dominant model, Begg’s test, P = 0.531, Egger’s test, P = 0.542; allele model, Begg’s test, P = 0.805, Egger’s test, P = 0.601).

## 4. Discussion

Polymorphisms in *NOD2* have been associated with the risk of many cancers, including lymphoma, CRC, gastric cancer, breast cancer, ovarian cancer, lung cancer, and laryngeal cancer [9]. Although a number of genetic association studies have been conducted to investigate the association between *NOD2* polymorphisms and CRC risk, there is still no clear consensus on their role, as many studies have reported conflicting results. Therefore, in the present study, we performed a meta-analysis of 13 independent case–control studies that included 5,013 cases and 4,463 controls to evaluate the association of *NOD2* polymorphisms with CRC risk. The main finding of the present meta-analysis was that both rs2066845 and rs2066847 were associated with an increased risk of CRC under heterozygous, dominant, and allele genetic models. Interestingly, subgroup analysis by ethnicity revealed that rs2066847 was not associated with increased CRC risk in Caucasians but was significant in participants of other ethnicities. On the contrary, for the rs2066844 polymorphism, we found no significant association with CRC risk. Analysis of the rs2066842 polymorphism was not possible because the allele frequency of this polymorphism was very low, resulting in zero-count cells that did not allow calculation of the pooled data.

The rs2066842 polymorphism involves an amino acid change from proline to serine, but it has been speculated to have no adverse effects on the protein function [29]. For this reason, previous studies conducted in the German and New Zealand Caucasian populations have reported that the rs2066842 polymorphism was not associated with the risk of gastric cancer and CRC, respectively [19,30]. It was also found that the polymorphism was not able to alter gene function when assessed alone [31]. Moreover, unlike the other three *NOD2* polymorphisms, this polymorphism was located neither in between nor within the key protein domains of NOD2 and therefore could not directly trigger NF-κB activation in response to bacterial lipopolysaccharide and peptidoglycan [32]. Nevertheless, the rs2066842 polymorphism has been described to have a protective effect against CD in the Arab population of Kuwait and against tuberculosis in the African American population [33,34]. In another study by Szeliga et al. [22], it was found that the T allele of this polymorphism may be associated with a higher risk of rectal cancer in the Polish population. In these cases, it is postulated that the polymorphism was in linkage disequilibrium (LD) with other causal variants that caused the disease, resulting in the presence of disease associations in some populations studied [35]. 

In addition, although previous studies on gastric carcinoma [36], gastric lymphoma [37], glioblastoma [38], and CRC [19,21] have all shown significant associations with the rs2066844 polymorphism, these results were not reflected in this meta-analysis. We found no significant association between this polymorphism and the risk of CRC, which is consistent with studies reported on other diseases, including malignant melanoma and gastrointestinal diseases [39,40,41]. In contrast to the location of the rs2066845 and rs2066847 polymorphisms, which are located in the leucine-rich repeat (LRR) region and contribute to a loss-of-function phenotype, rs2066844 is positioned between the LRR and nucleotide-binding domains [42]. Given this position, rs2066844 is most likely innocuous and may not affect responses to MDP and/or downstream signaling pathways, explaining our observation of the lack of significant association [43]. Nevertheless, there is a possibility that the polymorphism has a minor effect or may co-occur with other polymorphisms to alter NOD2 protein function, explaining why a significant association was observed in several cancers [44].

In addition, we demonstrated that the *NOD2* rs2066845 polymorphism was significantly associated with CRC risk under all genetic models analyzed. This result contrasted with that observed in Germany, Hungary, Portugal, and Finland [17,18,20,45]. Nonetheless, there are also studies showing significant associations between the rs2066845 polymorphism and the risk of CRC [21] and other diseases, such as pangastritis [46], CD [47], pulmonary non-tuberculous mycobacterial infections [48], and sarcoidosis [49]. These significant associations could be explained by the location of the polymorphism in the LRR domain, which mediates the protein–ligand interaction of NOD2. In fact, the positive association of this polymorphism was reaffirmed by a functionality assessment, which showed that the polymorphism may have deleterious effects on the function of the receptor [50]. Moreover, it was bioinformatically predicted that rs2066845 may cause impairment to the protein structure [50], further justifying our observation that the polymorphism was significantly associated with CRC risk.

Finally, rs2066847 is perhaps the most studied polymorphism among all *NOD2* polymorphisms. This polymorphism involves a cytosine insertion that results in a premature stop codon and thus LRR domain truncation [44]. Consequently, the variant allele is incapable of stimulating an appropriate response to activate the NF-κB, as it can only recognize lipopolysaccharide instead of MDP [51]. All the four polymorphisms are well known for their association with Crohn’s disease, which is characterized by defective innate immune responses and dysregulated intestinal inflammation. As mentioned above, these polymorphisms can impair the ability of NOD2 to recognize MDP, leading to compromised activation of the NF-κB signaling pathway. This defect disrupts epithelial barrier function and impairs bacterial clearance, contributing to persistent intestinal inflammation. Chronic inflammation, as seen in Crohn’s disease, is a known risk factor for colorectal cancer, and this inflammatory microenvironment may provide a biological basis for the observed association between *NOD2* polymorphisms and increased CRC risk.

In our study, we observed a significant association of the rs2066847 polymorphism with CRC risk. This result is consistent with the findings reported by several other studies on different types of solid tumors such as breast, lung, and gastric cancers, as well as non-Hodgkin’s lymphoma [39,52,53,54]. In addition, [11] concluded in their study of 12 different cancers that the lifetime risk of cancer increases by around 25% to 35% in the presence of the rs2066847 polymorphism. It has also been suggested that rs2066847, unlike the rs2066844 and rs2066845 polymorphisms, causes greater life-threatening disease progression due to its frameshift mutation in the LRR region, which plays an important role in immunological modulation [55]. However, there are also studies showing that this polymorphism is not associated with the risk of multiple myeloma and lung cancer in the Turkish population [44,56]. Thus, with all the controversial published findings, it is reasonable to assume that every key aspect, including geographic variability, source of control, prevalence of polymorphisms in specific populations, genotyping methods, differences in sample size, ethnicity, environmental and genetic factors, complex gene–gene or gene–environment interactions, and even mere chance, could play an important role in determining how these polymorphisms affect the development of disease risk [2,13,57]. 

Several limitations to this meta-analysis should be noted. First, the effects of gene–environment interactions could not be effectively assessed due to the limited number of studies that reported on this aspect. Second, for the rs2066842 polymorphism, only a small number of studies was included in the analysis and its allele frequencies were very low. This resulted in cells with null values, so quantitative data synthesis was not possible. Third, ethnicity was also not proportionally distributed in the included studies, as the majority of individuals analyzed for rs2066847 belonged to the “other ethnicity” subgroup, and only one of the included studies was conducted in Asia. Ethnic variation can influence the results of genetic association studies in different ways. For example, linkage disequilibrium patterns may be different among ethnic groups, meaning that a polymorphism may be in LD with a causal variant in one population but not in another. In addition, the effect size or direction of the association may be influenced by gene–environment and gene–gene interactions; thus, different ethnicities, which may have distinct genetic backgrounds, environmental exposures, lifestyle factors, and microbiome compositions, could exhibit variable risk profiles for the same polymorphism. To address the influence of ethnic variation in genetic association, future studies should include diverse ethnic groups, especially the underrepresented populations, in order to ensure a more comprehensive and generalizable assessment. Another limitation of this paper is that although we significantly improved the statistical power of the study, the sample size may still be too small to reliably assess the risk association. Finally, the certainty of the evidence was not formally assessed using a structured framework such as GRADE. Despite these limitations, our meta-analysis had several strengths. For instance, the quality of the included studies in this meta-analysis was considered high and met our inclusion criteria as presented in the Newcastle-Ottawa Scale. In addition, we did not find any publication bias in this meta-analysis, especially for the two polymorphisms that showed an association with the risk of CRC (rs2066845 and rs2066847). This means that the pooled results were unbiased.

## 5. Conclusions

The results of this meta-analysis showed that *NOD2* rs2066845 and rs2066847, but not *NOD2* rs2066842 and rs2066844, are associated with CRC risk. Interestingly, when stratified by ethnicity, the association of rs2066847 proved significant only in participants of “other ethnicities”, but not in Caucasians. However, the “other ethnicities” subgroup was itself a mixture of many different ancestries, and the small sample size did not allow us to further subdivide the subgroup into more specific ancestries. Therefore, future large-scale studies in different ethnicities are needed to obtain a convincing result on the influence of *NOD2* polymorphisms on CRC risk. Nevertheless, the current results showed that the rs2066845 and rs2066847 polymorphisms can potentially serve as predisposition biomarkers for CRC, although further validation work is needed.

## Figures and Tables

**Table 1:** Main characteristics of included studies.

Study	Year	Country	Ethnicity	Total Subjects	Genotype (Case/Control)			HWE (P Value)
				(Case/Control)	Wild-Type	Heterozygous	Variant	
**rs2066844**								
Burada [5]	2018	Romania	Other	108/265	100/247	8/18	0/0	0.567
Lakatos [18]	2007	Hungary	Caucasian	194/200	187/194	7/6	0/0	0.829
Lau [12]	2014	Malaysia	Asian	130/212	130/212	0/0	0/0	NA
Mockelmann [20]	2009	Northern Germany	Other	1,044/724	941/658	99/65	4/1	0.643
Papaconstantinou [21]	2005	Greek	Other	104/100	94/98	10/2	0/0	0.919
Roberts [19]	2006	New Zealand	Caucasian	133/201	116/189	15/12	2/0	0.662
Szeliga [22]	2008	Polish	Other	51/100	45/96	6/4 *		NA
Tuupanen [17]	2007	Finland	Caucasian	954/508	913/487	40/21	1/0	0.634
**rs2066845**								
Lakatos [19]	2007	Hungary	Caucasian	194/200	187/193	7/7	0/0	0.541
Lau [12]	2014	Malaysia	Asian	130/212	130/212	0/0	0/0	NA
Mockelmann [20]	2009	Northern Germany	Other	1,044/724	1,014/707	29/17	1/0	0.749
Papaconstantinou [21]	2005	Greek	Other	104/100	86/93	18/7	0/0	0.716
Roberts [19]	2006	New Zealand	Caucasian	133/201	127/198	6/3	0/0	0.915
Szeliga [22]	2008	Polish	Other	51/100	49/98	2/2 *		NA
Tuupanen [17]	2007	Finland	Caucasian	960/508	954/506	6/2	0/0	0.964
**rs2066847**								
Alhopuro [24]	2004	Finland	Caucasian	926/348	892/335	33/13	1/0	0.722
Kurzawski [25]	2004	Poland	Other	300/300	263/279	37/21 *		NA
Lakatos [18]	2007	Hungary	Caucasian	194/200	180/190	14/10	0/0	0.716
Lau [12]	2014	Malaysia	Asian	130/212	130/212	0/0	0/0	NA
Mockelmann [20]	2009	Northern Germany	Other	1,044/724	972/686	69/36	3/2	0.045
Omrane [26]	2014	Tunisia	Other	101/145	99/143	2/2	0/0	0.933
Papaconstantinou [21]	2005	Greek	Other	104/100	78/88	26/12	0/0	0.523
Roberts [19]	2006	New Zealand	Caucasian	133/201	127/197	6/4	0/0	0.886
Suchy [27]	2008	Poland	Other	607/607	544/558	60/49	3/0	0.300
Szeliga [22]	2008	Polish	Other	51/100	46/93	5/7 *		NA
Vogel [28]	2007	Danish	Other	355/753	344/728	11/25	0/0	0.643
**rs2066842**								
Lau [12]	2014	Malaysia	Asian	130/212	126/204	4/8	0/0	0.779
Roberts [22]	2006	New Zealand	Caucasian	133/201	86/109	37/80	10/12	0.593
Szeliga [22]	2008	Polish	Other	51/100	24/70	27/30 *		NA

* Reported the combined frequencies of heterozygous and variant genotypes.

**Table 2:** Newcastle-Ottawa Scale for assessing the quality of the included studies.

Study	Selection				Comparability	Exposure			Total Star
	Criteria				Criteria	Criteria			
	1	2	3	4	1	1	2	3	
Vogel et al. [28]	✭	✭	✭		✭✭		✭	✭	7
Mockelmann et al. * [20]	✭	✭	✭/0	✭	✭✭		✭	✭	7/8
Lakatos et al. [18]	✭	✭	✭	✭	✭✭		✭	✭	8
Suchy et al. [27]	✭	✭		✭	✭✭		✭	✭	7
Burada et al. [5]	✭	✭		✭	✭✭		✭	✭	7
Szeliga et al. [22]	✭	✭		✭	✭✭		✭	✭	7
Lau et al. ^+^ [12]	✭	✭	✭/0	✭			✭	✭	5/6
Omrane et al. [26]	✭	✭		✭	✭✭		✭	✭	7
Tuupanen et al. [17]	✭	✭	✭	✭	✭✭		✭		7
Alhopuro et al. [24]	✭	✭	✭	✭	✭✭		✭		7
Kurzawski et al. [25]	✭	✭		✭	✭✭		✭	✭	7
Papaconstatinou et al. [21]	✭	✭		✭	✭✭		✭	✭	7
Roberts et al. [19]	✭	✭	✭	✭	✭✭		✭	✭	8

* One star in Criterion 3 for rs2066844 and rs2066845 (giving a final rating of 8 stars), but no star for rs2066847 due to deviation from the HWE (giving a final rating of 7 stars). The study did not examine rs2066842. ^+^ One star in Criterion 3 for rs2066842 (giving a final rating of 6 stars), but no star for the other three polymorphisms due to deviation from the HWE (giving a final rating of 5 stars).

**Table 3:** Association between *NOD2* rs2066844 polymorphism and colorectal cancer risk.

Contrast Model	Number of Studies	Number of Cases	Number of Controls	Model	OR (95% CI)	*P*_OR_
Heterozygous model						
Overall	6	2530	1997	Fixed	1.176 (0.922–1.501)	0.191
Caucasian	3	1278	909	Fixed	1.258 (0.832–1.903)	0.277
Other ethnicity	3	1252	1088	Fixed	1.135 (0.840–1.534)	0.408
Dominant model						
Overall	7	2588	2098	Fixed	1.253 (0.989–1.589)	0.062
Caucasian	3	1281	909	Fixed	1.329 (0.882–2.003)	0.174
Other ethnicity	4	1307	1189	Fixed	1.217 (0.910–1.627)	0.186
Allele model						
Overall	6	5074	3996	Fixed	1.243 (0.983–1.571)	0.069
Caucasian	3	2562	1818	Fixed	1.392 (0.932–2.079)	0.106
Other ethnicity	3	2512	2178	Fixed	1.172 (0.878–1.564)	0.281

**Table 4:** Association between *NOD2* rs2066845 polymorphism and colorectal cancer risk.

Contrast Model	Number of Studies	Number of Cases	Number of Controls	Model	OR (95% CI)	*P*_OR_
Heterozygous model						
Overall	5	2434	1733	Fixed	1.544 (1.014–2.349)	0.043
Caucasian	3	1287	909	Fixed	1.557 (0.735–3.297)	0.248
Other ethnicity	2	1147	824	Random	1.690 (0.744–3.835)	0.210
Dominant model						
Overall	6	2486	1833	Fixed	1.561 (1.035–2.354)	0.034
Caucasian	3	1287	909	Fixed	1.557 (0.735–3.297)	0.248
Other ethnicity	3	1199	924	Fixed	1.562 (0.956–2.552)	0.075
Allele model						
Overall	5	4870	3466	Fixed	1.572 (1.040–2.375)	0.032
Caucasian	3	2574	1818	Fixed	1.547 (0.734–3.261)	0.251
Other ethnicity	2	2296	1648	Fixed	1.583 (0.964–2.599)	0.069

**Table 5:** Association between *NOD2* rs2066847 polymorphism and colorectal cancer risk.

Contrast Model	Number of Studies	Number of Cases	Number of Controls	Model	OR (95% CI)	*P*_OR_
Heterozygous model						
Overall	8	3457	3076	Fixed	1.321 (1.060–1.647)	0.013
Caucasian	3	1252	749	Fixed	1.245 (0.772–2.008)	0.369
Other ethnicity	5	2205	2327	Fixed	1.343 (1.047–1.722)	0.020
Dominant model						
Overall	10	3815	3478	Fixed	1.402 (1.147–1.713)	0.001
Caucasian	3	1253	749	Fixed	1.264 (0.784–2.036)	0.337
Other ethnicity	7	2562	2729	Fixed	1.434 (1.149–1.788)	0.001
Allele model						
Overall	8	6928	6156	Fixed	1.345 (1.088–1.663)	0.006
Caucasian	3	2506	1498	Fixed	1.276 (0.797–2.043)	0.311
Other ethnicity	5	4422	4658	Fixed	1.364 (1.076–1.729)	0.010

## Data Availability

Data is contained within the article or Supplementary Material. The review protocol is available from the corresponding author upon reasonable request.

## Supplementary Materials

The following supporting information can be downloaded at: https://www.mdpi.com/article/10.3390/cancers17121999/s1, Figure S1: Sensitivity analysis of *NOD2* rs2066844 and colorectal cancer risk.
