# Supplementary material for: NOD2 Polymorphisms and Their Association with Colorectal Cancer Risk: An Updated Systematic Review and Meta-Analysis
Source: Cancers (Basel). 2025 Jun 15;17(12):1999. doi: 10.3390/cancers17121999 (PMC12190208; doi:10.3390/cancers17121999)
Supplement: Supplementary file 1 [file cancers-17-01999-s001.zip › cancers-3685161-supplementary.pdf]

Additional file

A) Homozygous model

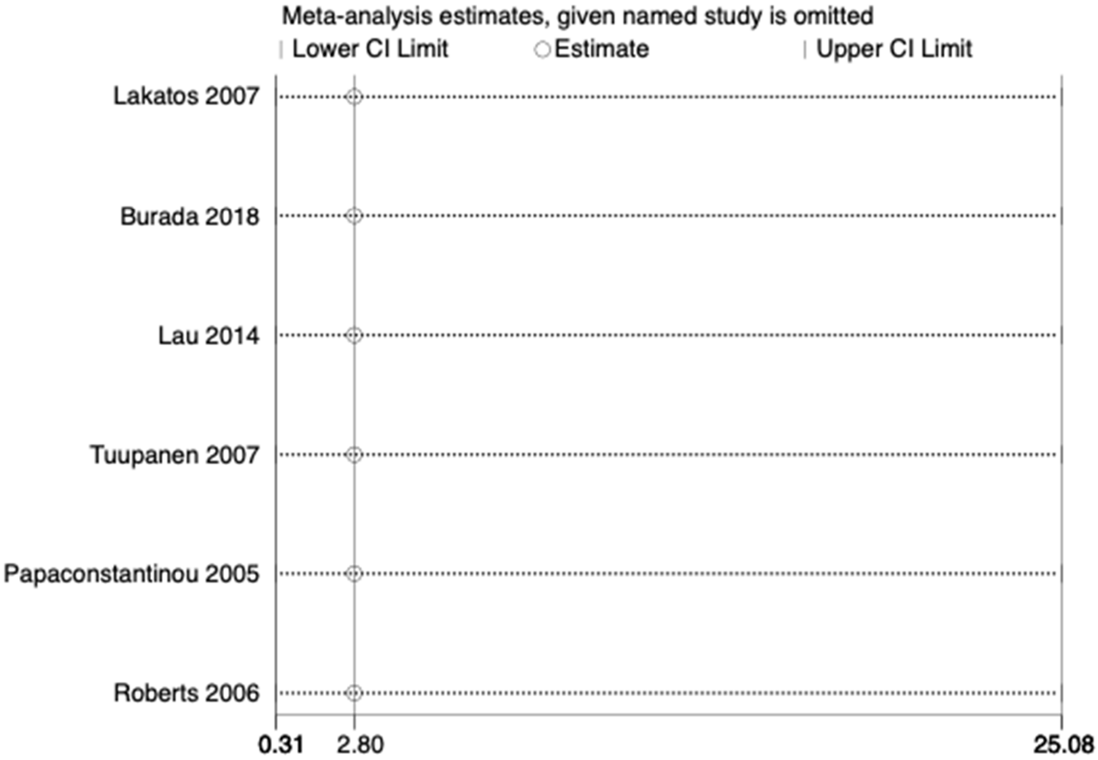

B) Heterozygous model

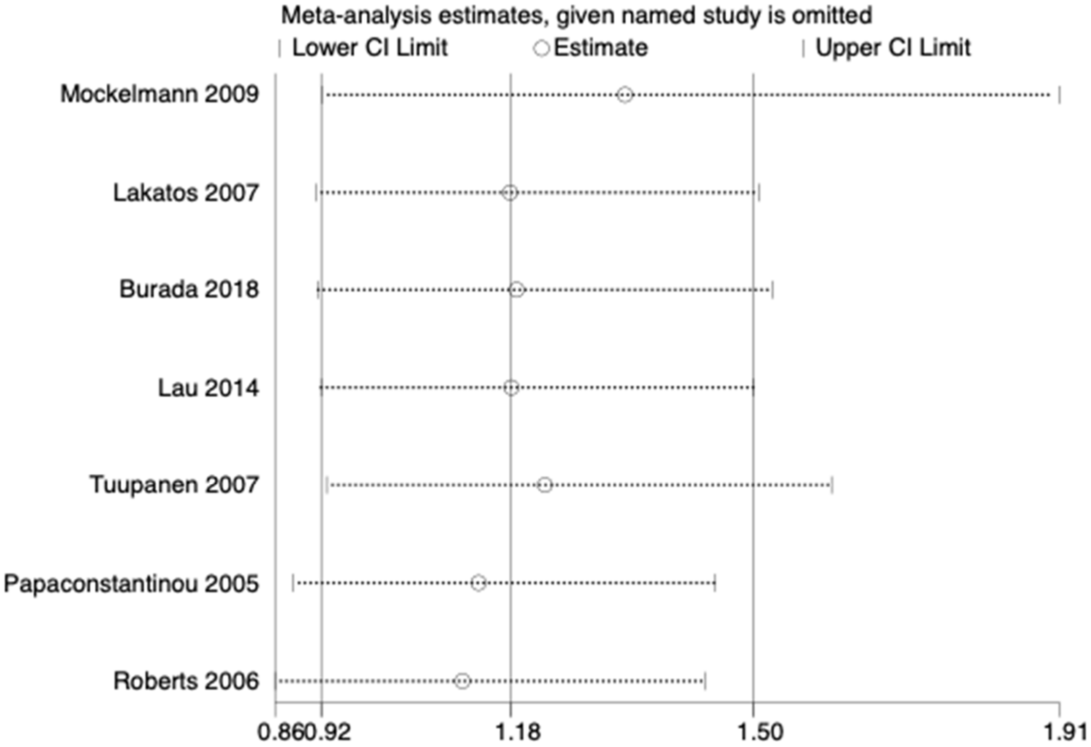

C) Dominant model

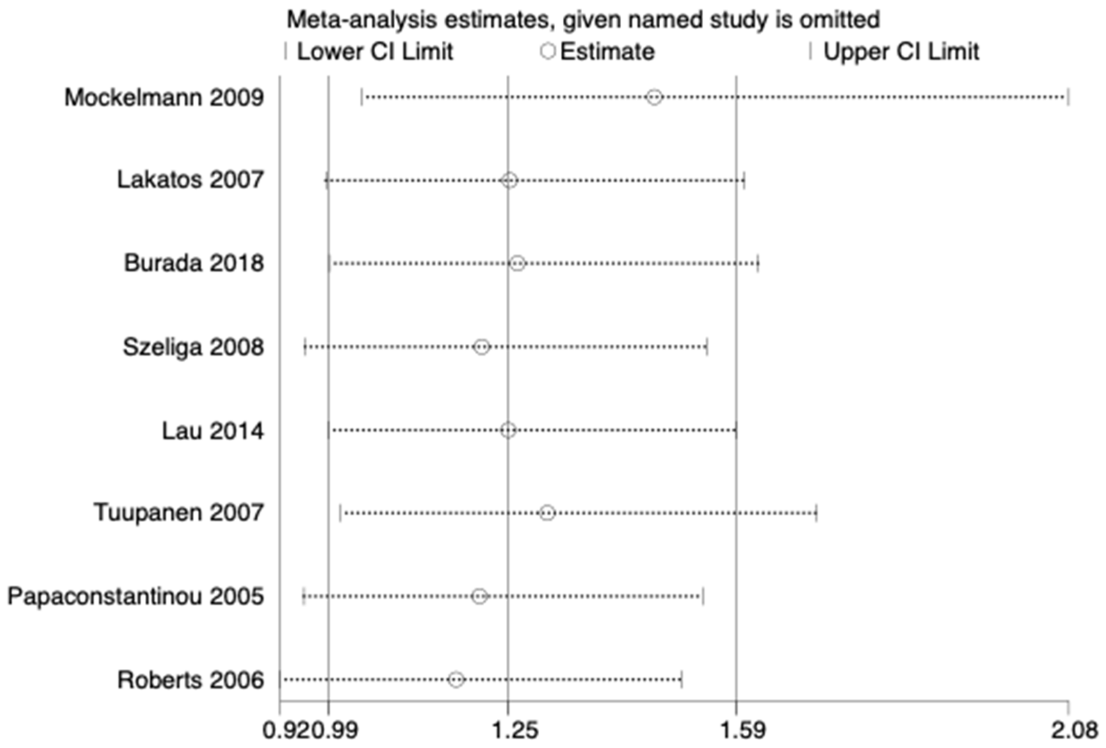

D) Recessive model

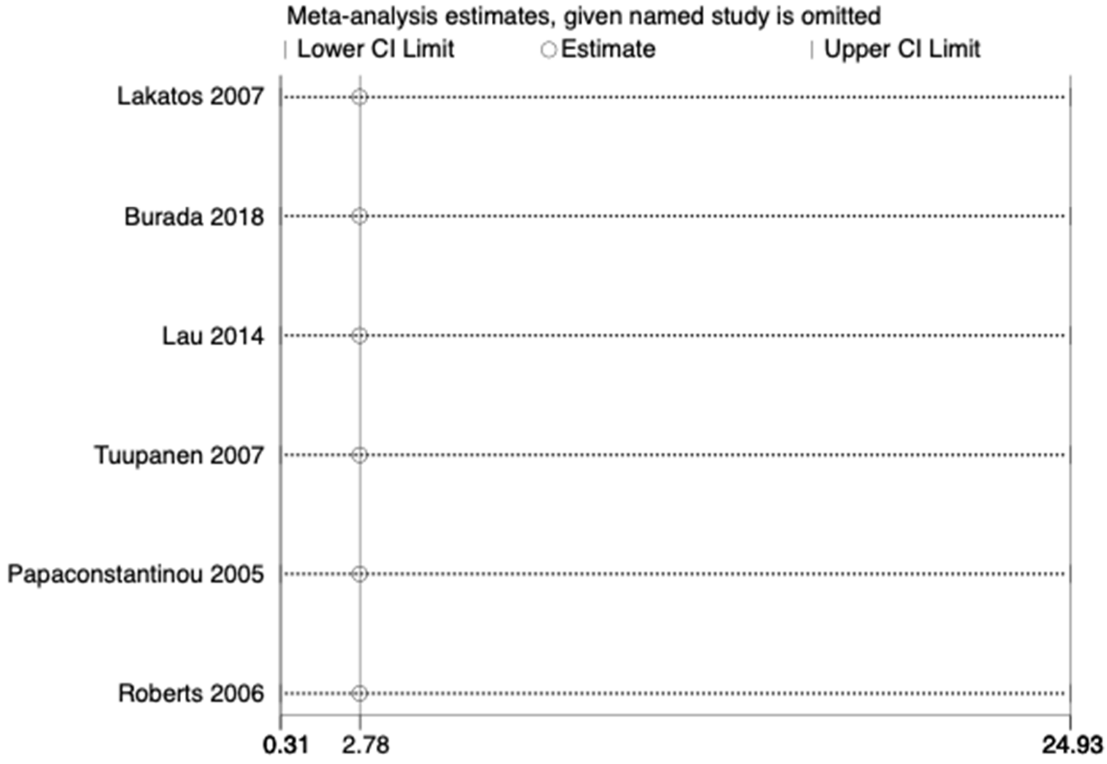

E) Allele model

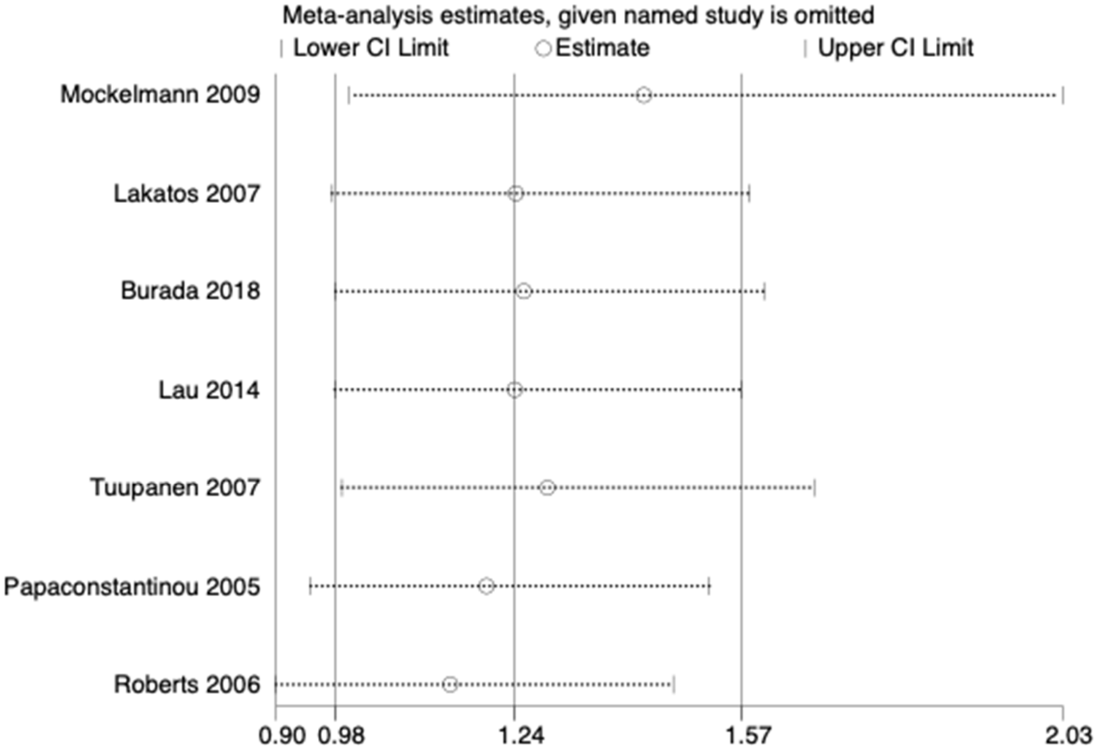

Figure S1. Sensitivity analysis of *NOD2* rs2066844 and colorectal cancer risk
